# Looking inside the Blackbox: Cuenca’s water metabolism

**DOI:** 10.1371/journal.pone.0273629

**Published:** 2022-09-22

**Authors:** Antonio Malo-Larrea, Vinicio Santillán, Esteban Torracchi-Carrasco

**Affiliations:** 1 Carrera de Educación en Ciencias Experimentales-Universidad Nacional de Educación (UNAE)—Av. Independencia s/n-Azogues, Chuquipata Centro, Ecuador; 2 Institut de Ciència i Tecnologia Ambientals (ICTA-UAB), edifici ICTA-ICP (Z), Bellaterra, Barcelona, Spain; 3 Centro de Investigación, Innovación y Transferencia de Tecnología (CIITT)-Universidad Católica de Cuenca, Vía Bibín-Cuenca, Cuenca, Ecuador; 4 Carrera de Odontología, Unidad Académica de Ciencias de la Salud-Universidad Católica de Cuenca, Cuenca, Ecuador; Universitat Autonoma de Barcelona, SPAIN

## Abstract

The socio-ecological metabolism of the water connects concepts that emerge out of the complexity of ecosystems, linking endosomatic processes that are indispensable for society while forming different hierarchic levels and relations among as well as aligning to their particularities. The consumptive uses of Cuenca city in their different categories and social metabolism both at rural and urban levels were assessed, inquiring about diverse typologies of the city’s water. Water Metabolic Rates (WMR) were calculated for each one of the consumptives’ uses gauged in liters per hour of human activity. Our results indicate that farming and industrial uses of water were highly inefficient. Linked to farms, both consumption of water and metabolic rates were higher in the rural areas. While paid work showed higher metabolic rates than households. Rural households evidenced a greater use of water and higher metabolic rates than urban households as water use combines human consumption and family farming. This research determined the water metabolism of the socio-ecological system in the canton of Cuenca, Ecuador through different dimensions of water metabolism. Formulating a system of flows and uses of water that were metabolized by different hierarchy levels of diverse consumptive uses within the aforementioned canton, as a tool to implement policies that guarantee water access and ecological metabolism, linking social dynamics within ecosystems.

## Introduction

Ecological Metabolism refers to how ecosystems operate from the perspective of complex systems [1], studying higher levels of system hierarchy such as populations, communities, or ecosystems [1, 2]. Ecological Metabolism incorporates cellular systems or atomic structure (i.e., bacteria), genetic-social systems (i.e., some plants), learning-organism systems (i.e., some mammals), and self-conscious organisms (i.e., humans) [2]. Ecological metabolism has connotations that are beyond mere flows and the processing of energy and matter and implies that evolution affects not only individuals but also the systems as units [3]. Regarding ecosystems as open systems and the fact that their metabolism involves energy, information, and matter entrance and exit in all biological levels [1, 2], from a single cell to the ecosphere, which includes social and ecosystem metabolism [4].

Social metabolism links both social and ecological systems [4] through a group of processes about human societies appropriate from the ecological metabolism and its own processes through circulation, transformation, consumption, and excretion of its different products (energy, matter, and information [5]). Human societies can be understood as complex, organic, and dynamic systems [6, 7] that produce and reproduce the vital conditions for their existence drawing from their metabolism with nature [5]. Stability and upkeeping functions and infrastructures of social systems depend on their metabolism, borrowing low-entropy matter, energy, and information, which are then returned to the ecosystem at high entropy [8–10]. Therefore, the ecological system regulates how societies are determined, and the organization of these societies determines the transformations of the ecological system [5]. Social metabolism is crucial in order to understand the socio-ecological process of coevolution, mirroring the adaptation of human beings to an environment while readapting to it and actively transforming this environment [11–13]. Social metabolism is considered an ecological-historical process, on the basis of what is social itself, through which societies’ endurance is rooted [14]. Nonetheless, the organic point of view of social metabolism by itself does not explain the historically produced space, does not include dynamic processes of social and political struggle, and does not state the dialectics of relations between culture and nature that mutually constitute the human space [6]. Social metabolism is both an organic process that implies appropriation, circulation, transformation, consumption, and excretion of matter, information, and energy [5]; and a dialectic, ecological-historical process that shapes the idea of nature in the social consciousness and the creation of environmental and territorial policies [14, 15]. Consequently, social metabolism is a socio-ecological and historical process that allows for the formulation of political questions about nature and the environment [14].

Urban areas generally are perceived as the places where nature ends and artificial begins, which reflects in the policies that rule them [16]. However, cities are ecological entities and are a physical manifestation of socio-ecological metabolism, where different ecological, socio-cultural, political, economic, and infrastructural dimensions collide [17]. Cities are places where ecological and social relations can be understood as part of nature [16]. They can be structured and formed by networks of socio-ecological processes, which simultaneously are global, local, ecological, human, material, discursive, cultural, and organic [13, 18]. Cities are places where liberating activities are performed and where wishes are materialized, yet at the same time, they are spaces where domination, systematic power, dangers, oppression, and exclusion exist [19]. Conflicts and compromises related to urban sustainability orbit around wide socio-ecological networks, in which urbanization is submerged [14, 16]. This is a socio-ecological process of metabolization of the ecological system [20].

Therefore, it is crucial to understand the city as a social perception, which is arbitrarily constructed based on the urban-rural dichotomy that is present in many various disciplines related to different scientific and historical discourses [18]. Urban metabolism is a crucial factor to understand urban-rural relations as cities are both the brain and the parasite of territories [21, 22]. This concept is intrinsically linked with rural metabolism, which is the result of spaces that are extremely heterogeneous, unable to be explained by the stereotypical idea of the urban-rural dichotomy [18]. What is urban and rural must be understood as socio-ecological systems that depend on agriculture, farming, forestry, hunting, fishing, recollection, and extraction for their survival [5]. That is how specifically cities and social systems, in general, are transformed into socio-natural hybrids that are behind the production of new environments, through cultural-social-political-economical dynamics. These dynamics are fundamental for the reproduction of society and to guarantee its quality of life [23].

This socio-ecological system depends on water to remain and reproduce [24]. Water is a fundamental element for the metabolism of societies due to being part of every endosomatic process of human activity, as well as being indispensable for most of the processes of every day [25]. Topics related to water have been traditionally addressed using criteria predominantly based on engineering, economy, or management, and less addressed through political, social, and environmental matters [20]. For example, water scarcity has been understood more as a natural process than a process that has been socially constructed, caused, and determined. The constant flow of water into, through, and out of a city upholds social systems [26] and makes life and human society’s essence constituting practices possible [20]. This circulation depends on technological networks that constitute water infrastructure, therefore guaranteeing adequate quality and provision of the liquid [6] and pushing towards the development and growth of cities that surround the idea of the modern city [27]. As such, a process of social appropriation of water exists that emerges from ecological metabolism, determined by complex political, economic, social, cultural, and ecological processes [20, 28].

This study used a Multi-Scale Integrated Analysis of Societal and Ecological Metabolism (MuSIASEM) [29] in order to explore the social metabolism of water in Cuenca City, Ecuador. First, an analytic framework of flows and hydric uses that were being metabolized by different hierarchical levels was developed [30], while assessing different consumptive uses of drinking and non-billed water using different categories of use as defined by the *Empresa Pública de Agua Potable* (public city water company). Then, the social metabolism of water was identified within the territory, both urban and rural, inquiring on different typologies of water; calculating the water metabolic rates (WMR) for each one of the different uses of water measured in liters per hour of human activity. In this context, higher net uses of water and metabolic rates were expected in the rural areas, as they were linked to agriculture [31]. Also, a clear difference was expected between rural and urban homes, understanding that Cuenca City widely exceeds the international standards for water consumption [32, 33]. Finally, higher metabolic rates were expected within the manufacturing industry than in other sectors of urban paid work [34] with a great variation in the gross added value of urban paid work [7, 25, 35].

## Materials and methods

### Study area

**Fig 1:**
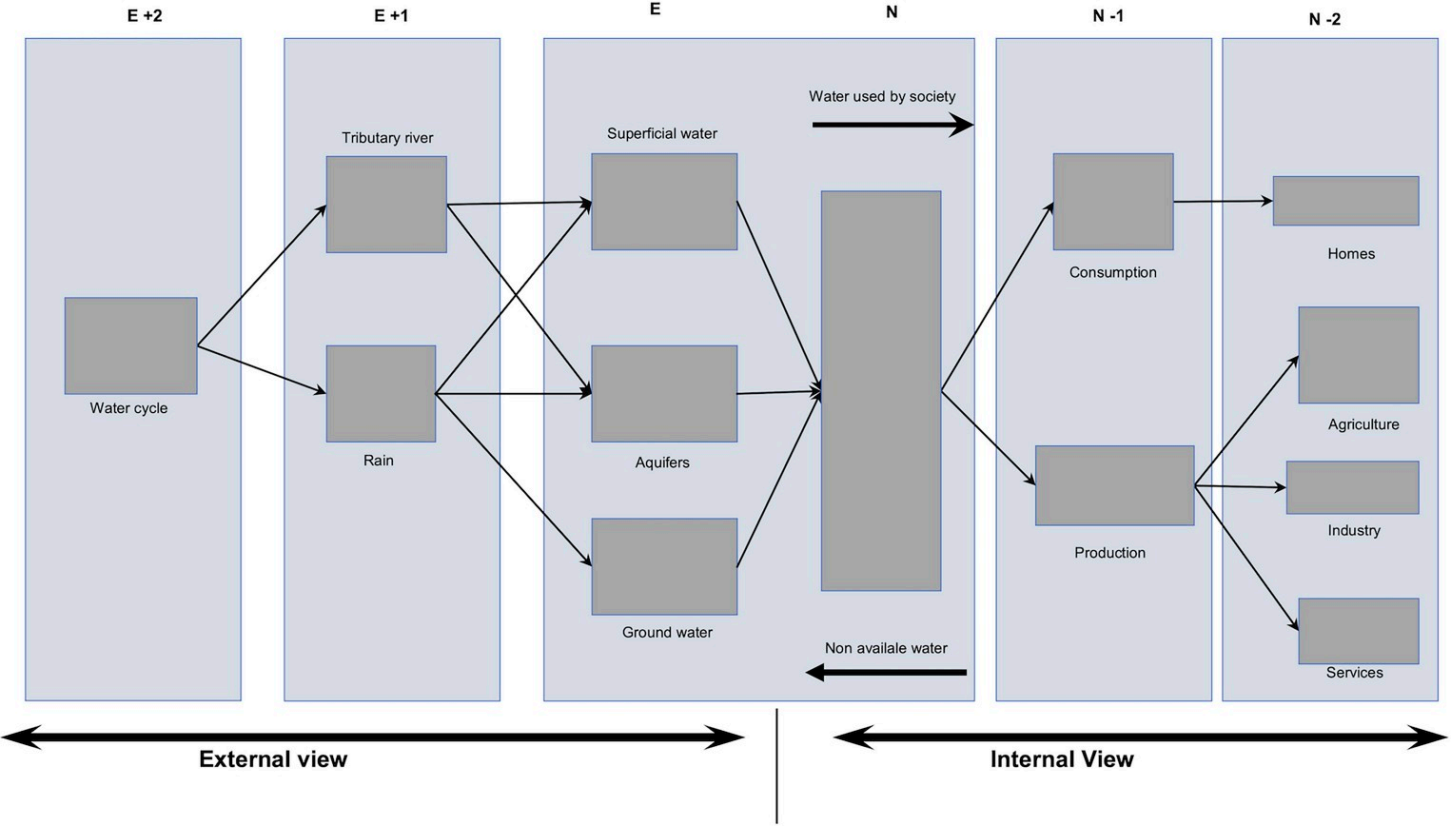
A map representation of Cuenca´s territory. Prepared: By the authors.

This study was carried out in Cuenca canton, located in the southern Andean region in Ecuador (Fig 1). This Andean canton belongs to *Macizo del Cajas*, a geologic formation delineated by the deep canyons of Cañar river and Jubones river, which develop into the Pacific Ocean. The territory of the canton lies both toward the Amazon and the Pacific watersheds with an approximate surface of 3190 km^2^. This complex territorial system ranges from the high Andes at an altitude of 4525 masl to the plain of lower lands in the Pacific Ecuadorian coastal region at 30 meters above sea level. The Amazon watershed area extends in the inter-Andean valley to 2315 masl where the urban area is located at altitudes between 2850 and 2335 masl.

### Ecologic system and territory

The temperature (ranging from -2°C to 31.7°C) and amount of precipitation (664.38 to 1835.73 mm) illustrate the heterogeneity of Cuenca canton and its diversity of microclimates. Cuenca’s zones with greater precipitation levels are located towards the foothills of Pacific Ocean slopes, while southern Pacific zones and urban zones present lower precipitation levels. The highest river volume period starts in March and ends in June, while the low river volume period starts in August and ends in October, while slightly dropping again between December and January. The variation in river volume is directly related to the rainy seasons. May and April are the months where the highest precipitation levels are present, followed by February, March, and October. The lowest precipitation months are August, July, and September, followed by December, January, November, and June [36]. *El Niño* phenomenon strongly affected the country in 1997 and 1998 (El Niño/Southern Oscillation-ENSO), and during 2003 and 2004 it moderately affected the region [37, 38] which affected rain seasons.

**Fig 2:**
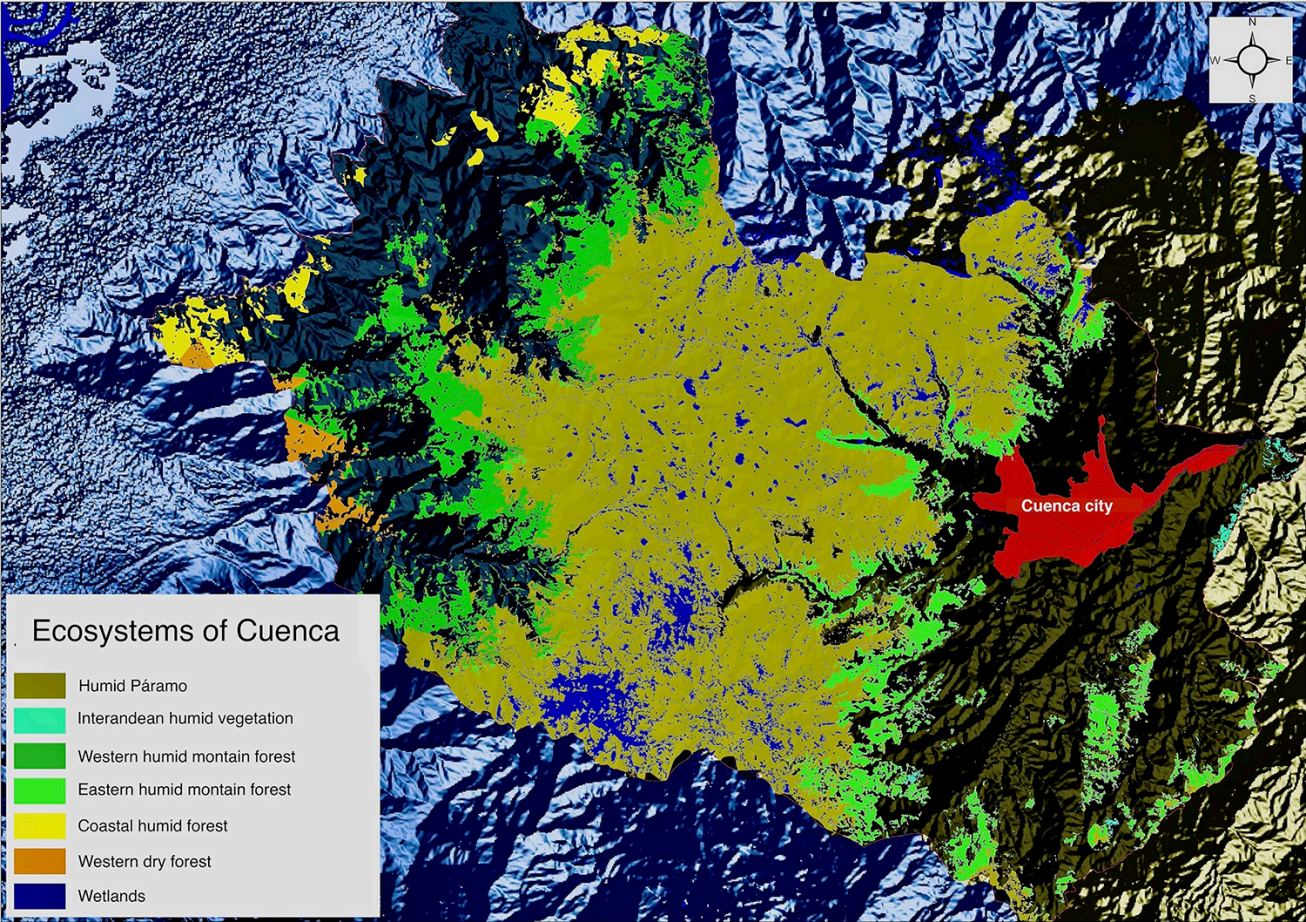
A map representation of the ecosystems identified in the territory of canton Cuenca. Prepared: By the first author.

The territory of Cuenca canton is located both in the Andean-Pacific watershed (43.6%) as well as in the Amazonian watershed (56.4%). Thirteen vegetation formations are grouped in seven ecosystems (tropical dry forest, tropical humid forest, western and eastern montane humid forest, inter-Andean vegetation, and paramo [39]) (Fig 2). This transformed landscape represents 38.93% of the territory. Cajas National Park, Quimsacocha National Recreation Area, and eight different protecting woodlands are located within Cuenca canton, all covering six of the aforementioned seven ecosystems excluding inter-Andean rain vegetation [39]. Cajas National Park is the core of the *Macizo del Cajas* biosphere’s reserve, it is a wetland of international importance RAMSAR and Important Bird Area IBA [40].

Among these eight identified ecosystems, 24 different land coverage have been defined for Cuenca canton. For this study, this land coverage has been grouped into eight groups: urban and infrastructure areas, crop areas, livestock areas, undetermined vegetation areas, native ecosystems, aquatic ecosystems, worsened areas, and areas lacking information. The undetermined vegetation areas cover 35.97% of the territory, 14.59% of the canton’s territory has been dedicated to livestock farming, and 6.02% has been dedicated to agriculture. Probably, because of irregular topography in the territory with steep slopes and very few amounts of available zones for livestock or farming. Nonetheless, these activities are widely distributed in its territory except for the highest zones, which are used mainly for subsistence even though consolidated agroindustry dedicated to dairy and flower production is present.

### The social and economic system

Cuenca city is the highest densely populated city in Ecuador, rating 47 inhabitants per hectare [41]. Its population is concentrated in the urban zone (65.6%). The working population represents 26% of the total amount of inhabitants and the rural population represents 34% of the total amount of working population. Cuenca is home to every production sector that is present in Ecuador [42]. ETAPA EP (*Empresa Pública de Teléfonos*, *Agua Potable y Alcantarillado*) owns three drinking water treatment plants and residual water treating plant that treats 88% of residual water in the city. The rivers Tomebamba, Yanuncay, Tarqui, and Machángara course through the urban zones which together form the Cuenca River, the main river tributary to the Paute River, which provides 30% of national electricity [39]. There are five concessions for electricity generation for *Corporación Eléctrica del Ecuador (CELEC)*, which must guarantee adequate water volumes for the correct operation of hydroelectric projects in the country [43].

The most pressured by agriculture sub-basins in the area are those of Tarqui River (36.2%), Machángara River (30.1%), and Tomebamba River (19.2%). The industry is generally concentrated in the sub-basin of Machángara River (45.7%) and Tomebamba River (39.2%, mainly stoneware production industry). The water availability for homes is concentrated in the Tomebamba River (74%) and Yanuncay River (12.6%). Hydroelectricity is mainly produced in the high basin of the Machángara River (85.7%) (S1 Table).

### Multi-scale analysis of societal and ecological metabolism of water

In order to study the socio-ecological metabolism of water in Cuenca, we used a Multi-Scale Integrated Analysis of Societal and Ecosystem Metabolism (MuSIASEM) [29, 35, 44]. MuSIASEM is based on the idea of a semantically open grammar [44–46], which means that the system can be metaphorized according to its own particularities [29, 45, 47]. Therefore, the analysis is not only able to be adapted to any system, but is also compatible with any representation of the system [45, 47, 48], allowing for every chosen level for the study to be incorporated and showing relations between elements while quantifying their metabolism among elements of the same level and between levels as well [13, 45, 46, 48].

The use of MuSIASEM focus in the study of water metabolism brings interesting challenges as water is both a fund and a flow at the same time [30, 45, 48–50]. Water as a molecule is a fund, as it remains unaltered during all of its metabolic process and can be seen from a macro point of view in the ecological system [30]. In contrast, the characteristics that determine the quality of water change during the metabolic process, therefore they can be considered a flow and, in that context, are related to what a society does [25].

**Fig 3:**
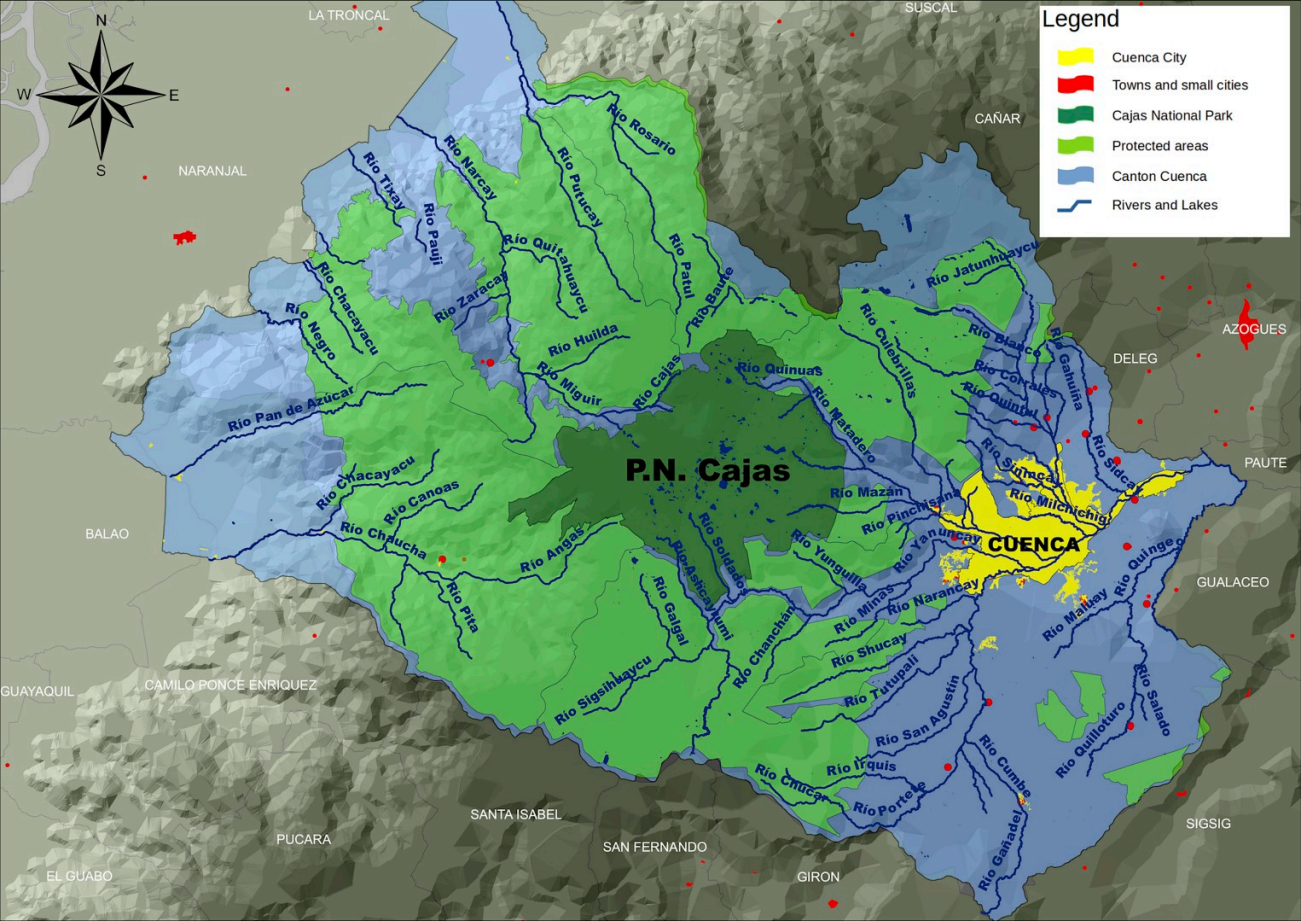
Outline of the metabolic pattern of water. It combines the organization in hierarchical levels of the metabolic pattern of society (N-i) with the ecosystem metabolic pattern (E-i). Adapted from Madrid & Giampetro (2014).

Madrid and Giampietro [30] represent MuSIASEM starting from an external view, *E* (Fig 3), that refers to blue water (superficial water and aquifers), green water (water stored in the soil, not available for human use but available for plants) and the water cycle in its entirety. The external view is based on the semantic category of Ecosystem Water Recharge (EWR) [30, 35]. EWR involves water entry in the form of blue water, as well as the processes of water cycles. The semantic category that links the ecological system and social system is the Social Appropriation of Water (SAW). SAW refers to the total amount of water extracted for human use and the changes that its quality experiences as it is metabolized by society [30, 35]. The internal view of MuSIASEM representation links the ecologic system and the social system (N Level) (Fig 3). The semantic category to analyze this level is Gross Water Use (GWU) which refers to the total volume of water being appropriated by society, including water that is not accounted for and losses [30, 35]. The internal view is represented by levels ranging from N-1 to N-i. Using both aforementioned semantic categories (quality level, losses during distribution process, non-measured water) and the Net Water Use (NWU, water that effectively reaches its final destination, categorized in different uses) [30, 35].

**Fig 4:**
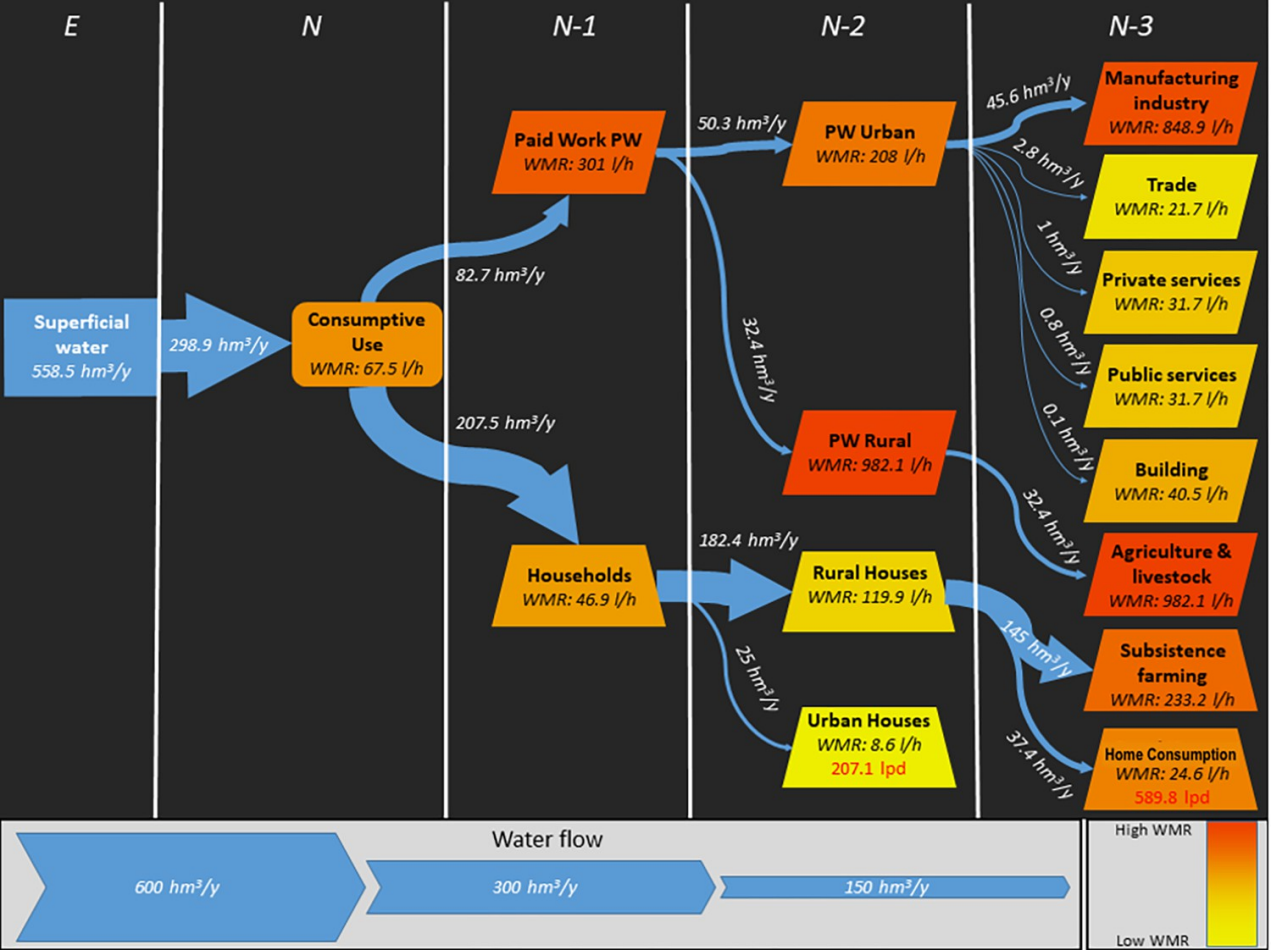
Representation of water metabolism of water (MuSIASEM) in Cuenca. Water flows are shown in cubic hectometers (106 cubic meters) and their metabolic rates in liters per hour. The Net Use of Water (NWU) was divided into paid work and homes (N-1). Next, these were subdivided into urban and rural uses (N-2). Finally, specific uses for each category were identified (N-3). The water flow for each category is represented in the thickness of each arrow and the metabolic rates are represented by a color map (Orange means high, yellow means low). Urban and rural home consumption is detailed in liters per day (lpd), in red.

The grammar used for the application of MuSIASEM in Cuenca was formed by levels in a dendrogram (Fig 4 detailed in the Results section). Level E refers to superficial water available for human appropriation (SAW). Levels N refers to the consumptive uses of water in Cuenca. Level N-1 groups paid work and homes. N-2 level refers to the urban sector and rural sector. Level N-3 refers to different sectors of paid work both at an urban (manufacturing industry, commerce, private services, public services, and government services as well as building) and a rural level (agriculture, livestock, silviculture, and fishing) and the subsistence agriculture for homes. Water available for Social Appropriation (SAW, Level E) represents the annual water volume of the Cuenca River which was calculated based on the median daily water volumes of the river (in 1997–2003 and 2008–2012). The annual volume of water purified by *ETAPA EP* was added to the resulting water volume. Also, 15.44% of water corresponding to environmental flow was deducted from the result. The calculations were held in this way since the gauging station of the water volumes of Cuenca River is located prior to the discharge point of Ucubamba Residual Waters Treatment Facility (PTAR), therefore excluding the addition from this facility. This water volume corresponds to the addition of the volume of purified water and rainwater gathered by sewage. The remaining volumes of wastewater are deposited with no treatment whatsoever directly in rivers and small streams. The wastewater is the result of water extracted for human consumption, normally retrieved from the same water body where water is taken from. As such, these residual waters were included in the water volume of the Cuenca River to calculate Level E.

Level N, which refers to Gross Water Use (GWU) in Cuenca, is the result of the addition of concessions for all uses given by former SENAGUA (i.e., former Secretaría Nacional del Agua or former Ecuadorian National Water Secretariat) (this institution was dissolved by the former Ecuadorian government) and the total volume of water purified by ETAPA EP. The Net Water Use was calculated at three different levels. Level N-1 separates the use of water in the paid work sector from the use of water in homes. Within the paid work sector, the use of water refers to that which was destined for industrial, commercial, agribusiness, public services, private services, and building sectors. These uses are destined by ETAPA EP and SENAGUA. Home use of water refers to the uses categorized as drinking water for domestic use. Level N-2 makes a difference between urban and rural sectors, regarding both home use and paid work use of water. The use of water in urban homes was the result of the addition of the residential use of water as stated by ETAPA EP, and the rural grants by SENAGUA. The rural consumption of water by rural homes refers to grants by SENAGUA for domestic use and exclusively for subsistence agriculture. Populations in Level N-2 homes were defined using National Housing and Population Census 2010 (CNPV-2021 [41]). In Level N-3, in order to calculate NWU of urban paid work, industrial grants by SENAGUA and the industrial category consumption as stated by ETAPA EP were added. It is worth noting that categories such as commercial use and building use, private services, public services, and government offices are managed by ETAPA EP.

Finally, hours of human activity were calculated with base on the National Survey on Employment, Unemployment and Subemployment 2012 (ENEDS-2012) annually held by INEC (National Institute of Statistics and Census). The calculation of the population involved in paid work was made based on the Economic Census of 2010, the last census of its kind held in Ecuador [42]. In order to calculate NWU of paid rural work different grants for agricultural use as defined by SENAGUA were grouped in a single agribusiness category. Additionally, data from CNPV-2010 was used to define the population working in paid agriculture.

Level N-3, rural homes, was linked to uses of water for subsistence agriculture and domestic use. NWU of subsistence agriculture was calculated based on agricultural metabolic rates. This population was estimated as 50% of the rural population, according to the results of the Continuous Surface and Agribusiness Production Survey in 2012. In order to define NWU of subsistence agriculture, we calculated the total water amount used by agriculture. Then, using the water metabolic rate for agriculture and livestock, we calculated the amount of water needed for this activity. We subtracted this data from the NWU of agriculture. The obtained result was the NWU of subsistence agriculture. In addition, we estimated the population performing subsistence agriculture. So as to, we subtracted the population working in paid agriculture from the total working in agriculture rural population. Finally, once we had obtained these two data, we were able to calculate subsistence agriculture Water Metabolic Rate.

Following the previously described grammar, the distribution of the population on different levels was analyzed, as well as the Social Appropriation of Water (SAW) by using Net Water Uses (NWU). Water represented by NWU is the flow, and people represented by hours of human activity is the fund [8], thus calculating the metabolic rate of the intensity of water use (WMR, expressed in liters per hour l/h) [30, 35]. On the one hand, the consumption of water at a household (home level) was calculated and estimated as the difference between Total Human Activity (8760 hours per year) and Paid work. On the other hand, paid work sector use was calculated by estimating eight hours of daily use (2080 hours per year). In order to calculate the metabolic rate of non-measured water a population of 331888 inhabitants was used (the urban population of Cuenca City) and 8760 hours of use per year. The fund-flow relation allows for a calculation of metabolic rates of a flow use, which then allows for comparisons between systems, between levels, and also between elements within the same system [30, 35]. Finally, the Economic Job Productivity (value-added per hour) was calculated for every different sector of paid work. This amount indicates higher efficiency as the value grows [25, 35].

## Results

Cuenca Canton has availability for Social Water Appropriation (SAW) of 558.5 hm3/year (MuSIASEM, Level E). Consumptive uses of water reach a Gross Water Use (GWU) of 298.9 hm3/year, and 2.9% of non-measured water. The Net Use of Water (NWU) reaches 290.2 hm3/year, with a Water Metabolic Rate (WMR) of 67.5 l/h (MuSIASEM, Level N). In level N-1, the net use of water (207.5 hm3/year) was greater than that of paid work sector (82.7 hm3/year). Despite that, the metabolic rate was greater for the paid work sector (301 l/h) than the metabolic rate of homes (46.9 l/h). In level N-2, paid work represented 16.8% of the net use of water for the urban sector, and 10.8% for the rural sector, with a higher metabolic rate for the rural sector (982.1 l/h) than for the urban sector (208 l/h). In rural homes, the net use of water was greater (61.1%) than in urban homes (8.4%). Despite that, in rural homes most of the use of water lies in non-paid agriculture or family agriculture (48.6%) as compared to consumption, which represented 12.5% of the use of water with metabolic rates that are higher for non-paid agriculture (233.2 l/h), followed by rural homes (24.6 l/h) and urban homes (8.6 l/h). Also, rural homes presented a greater consumption of liters of water per person per day (589.8 lpd) than urban homes (207.1 lpd). In level N-3, most of the net water use of water by paid work sectors is concentrated in the manufacturing industry (15.2%) with a metabolic rate of 848.9 l/h, and in agriculture and livestock (10.8%) with a metabolic rate of 982.1 l/h. Commerce, private and public services, and building showed a net use of water lower than 1% and metabolic rates in a range between 40.5 l/h and 21.7 l/h (Fig 4).

The urban sector had 25.2% of water consumption and in contrast, the rural sector holds 71.8%. In general, water consumption in homes represented 20.9% distributed in the following way: 8.4% for urban homes and 12.5% for rural homes. However, the consumption of water in rural homes is concentrated in non-paid or family agriculture (48.6%). Within paid work, the consumption of water for agriculture reached 10.8%. Agribusiness gathered 59.3% of the total water consumption of Cuenca (14.6% for livestock, 6.02% for agriculture). Industrial consumption of water represented 15.2% of total consumption and a great demand for water. The remaining production sector barely represented 1.54% of total water consumption.

Even though there were important differences between economic activities regarding efficiency in water use, it is fundamental to understand that these values must be compared with similar economic activities in order to create appropriate policies for each activity’s typology. Urban sector economic activities were more efficient than agribusiness concentrated in rural sectors (75.1 GVA/m3 and 0.1 GVA/m3, respectively). The rural sector, at the paid-work level, was represented by the agricultural, livestock, silviculture, and fishing category, understanding that category as agribusiness industries of different sizes. The most efficient sectors in the urban sector were commerce, followed by public services and building. The least efficient sectors were industries and private sectors (81.4 GVA/m3 and 84.4 GVA/m3 respectively) (Table 3). 84.4% of the Gross Added Value (GVA) in Cuenca was concentrated in wholesale and retail commerce (72.6%) and the manufacturing industry (11.8%). The former represented 13.74% of business establishments in the city, while the latter represented 47.01% of them. It is important to note that the Economic Census of 2010 is mistaken, as it states the city has a nonexistent cable industry. This mistake has led to a common belief that Cuenca is an industrial city. Once this error is corrected, it is clear that Cuenca is a business city and that the biggest business is car trading.

## Discussion

In the present study, an analytic framework was developed to assess the territory of Cuenca canton as a socio-ecological system. The water metabolism of its socio-ecological system was studied specifically, providing dimensions of water metabolism that surpass classic views on the matter. In order to do this, a system of flow and water uses that are metabolized by different hierarchical levels of consumptive uses of water in Cuenca was developed. A specific grammar was developed, constituting different organization levels and establishing two big sectors (paid-work and homes). Paid work was constituted by the main economic activities in Cuenca. Homes were divided into urban and rural categories. This study examined different categories of water use, evidencing the capacity of water metabolism for every consumptive use at distinct levels while identifying clear differences between what is urban and rural homes. Additionally, industrial and private service sectors were shown as the principal water consumers regarding water use in urban households.

The water grammar in Cuenca combines water semantics as either a flow or fund and defines levels and water classification in the same context [25, 35]. These semantics were used to provide such qualitative analysis parameters of metabolic patterns in MuSIASEM, which includes the use of descriptive domains to define analytic levels and classification of water that involves its roles as flow and fund and as a system [30, 50, 51]. A water grammar was designed to integrate water into the socioeconomic metabolism of Cuenca, defining water as a provision of a fund-flow [25, 50]. This means that the water volumes that are being extracted from structural ecosystem funds and bodies of water that must remain in a defined range of quantitative characteristics [52] locating narratives in the social metabolism space and integrating them as dendrograms within MuSIASEM grammars.

Regarding this described grammar, the distribution of population in its different levels, the social appropriation of waters (SQW), the gross use of water (GWU), and the net water use (NWU) were analyzed. According to the fund-flow model by Georgescu-Roegen [8], water that is represented by GWU and NWU are the flow, while people represented by hours of human activity are the fund. The relation between flow and fund allowed the calculation of metabolic rates (expressed in liters per hour l/h) of use of flow, which simultaneously allowed comparisons between systems in a way that would be impossible otherwise. In addition, comparisons between levels and elements within the same system, would not be possible using any other indicator [30]. A greater grant of water for human consumption than for productive uses [20, 24, 28, 30, 31] was demonstrated by identifying the distribution of NWU in Cuenca, that in contrast with previous studies. Nonetheless, these results were not consistent with the calculation of metabolic rates, that is, the efficiency of water for every hour of human activity.

The definition of levels and hierarchies evidenced a complexity level that implies epistemological issues at multiple scales [52–54]. Thus, this same system presented different identities depending on the scale of the observation [48]. The identity of an observed system is an entity (or individuality) different from its background and other systems that it interacts with [55]. As such, the identity of the system depended on a combination of relevant qualities that were selected (observable attributes) both for its perception as for its representation. The identity of the system was characterized by gauging the metabolic rates in each one of the observation levels [56]. The processes within each level acted as a filter for each of the processes in the lower levels in the hierarchy [13]. These hierarchic systems were represented not only in terms of holons, representing at the same time a part of the level and the totality formed by parts in lower levels [57]. Hierarchies can be expressed as a relation within levels and between levels among holons [13] and they could be defined as holarchies [58]. These relations should influence mutually and should also form cross relations [55]. Therefore, the holarchy of Cuenca shows an added behavior that does not equal the sum of the relations between each part, but one that emerges from them [59]. The exchange of information within a level operates at a rhythm that feeds from the rhythm of higher and lower levels, generating socio-ecological systems that are nested holarchies [13, 52] in which the social holons are a part of the ecosystem holons, therefore, expressing non-linear emerging properties autopoietic and dissipative behaviors [35].

Generally, the metabolic rates of Cuenca were very high in comparison to previous studies [49] which indicates high consumption of water, despite the fact that the absolute use of water in the canton is fairly low (Fig 4). The highest metabolic rates were presented in sectors such as agriculture, livestock, silviculture and fishing (agroindustry), industry, and subsistence agriculture. Urban homes and rural homes showed the lowest values. Nonetheless, the metabolic rates in these sectors were significantly high in comparison to Spain (Table 1) and Lima, Perú (Table 2) [49].

**Table 1:** Spain’s water metabolic rates.

N (l/h) (Average Society)	N-2 (l/h)		N-3 (l/h)	
0.06	Paid Work	0.68	Industry	0,82
			Agriculture	9,7
			Services	0,0084
	Homes	0.006		

Source: Madrid, Cristina, Violeta Cabello, y Mario Giampietro. 2013. «Water metabolism with MuSIASEM». Liphe4 Summer school presentation, Barcelona, Spain. http://prezi.com/j5avdvny8gor/musiasem4water_sp_school/.

**Table 2:** Lima’s, Perú water metabolic rates.

N (l/h) (Average Society)	N-1 (l/h)		N-3 (l/h)	
5.6	Paid Work	12.09	Industry	19.3
			Government	28.6
			Services	6.9
	Homes	4.7		

Source: Madrid, Cristina, Violeta Cabello, y Mario Giampietro. 2013. «Water metabolism with MuSIASEM». Liphe4 Summer school presentation, Barcelona, Spain. http://prezi.com/j5avdvny8gor/musiasem4water_sp_school/.

Water consumption per person per day (lpd) in Cuenca allowed us to better appreciate its magnitude, both for rural (589.8 lpd) and urban homes (207.1 lpd). At a national level, water consumption per person per day in Cuenca is higher than in Quito [60], but ranks much lower than Guayaquil [20]. At an international level, water consumption per person per day in Argentinian cities [61] and European cities [62] is much lower than that in Cuenca. Measurements in Cuenca exceed those recommended by the World Health Organization (100 lpd) [32] and those recommended by New Water Culture Foundation (120 lpd) [33]. Yet, the fact that water in rural homes is also used for the upkeeping of livestock, home orchards, and even the use of water for big-scale farming must be taken into account. Also, in rural homes, there is no potable water, but treated water that is assigned by local water commissions.

The general and total vision of MuSIASEM in Cuenca represents water flows, metabolic rates, and liters per day in urban and rural homes. Dendrograms representing these metabolic processes were used to explicitly relate fund and flow elements. A dendrogram is a diagram in the shape of a tree that shows taxonomic relations (classification) [63]. The amount of available water for the social system depends on water bodies’ recharge (focal level E). Thus, the recharge of water bodies is regulated by the ecosystem’s functions, channeling water supply as provided by the water cycle [2], taking into consideration that the appropriation of water bodies by parts of society allows for social functions to directly use water [20, 30]. In this study, a classification to differentiate the economically productive paid work and households in level N-1. In level N-2 primary productive activities and urban and rural homes. In level N-3 production-consumption in different paid work productive sectors, non-paid agriculture, and rural homes. All this allowed for the functional perception of funds, the classification of relevant flows, and the establishment of holon typologies [35, 50]. In this way, a dendrogram of funds and the taxonomy of the flow were developed (Fig 4). Identifying the processes that happen in Cuenca, at a social and ecosystem level. Therefore, the taxonomy here presented integrates all these dimensions and definitions of water, solving one of the key topics of the integrated management of hydric resources, the integration of water dimensions, and the assessment of water in metabolism studies [50].

An efficiency indicator for paid work was calculated by using the Economic Job Productivity (value-added per hour) (Table 3). This value indicates what added value is produced for each work hour, with higher values indicating higher efficiency [25, 34]. The most efficient sectors in generating this added value were commerce, followed by public services, building, industry, and private services. The notably least efficient sector was agribusiness which produces. It is important to note that the Economic Job Productivity of commerce doubled that of industry.

**Table 3:** Economic Job Productivity: Gross Value Added (GVA) per hour (US$/h). A higher number indicates higher efficiency.

*LEVEL (NWU)*	*SECTOR*	*GVA (US$)*	*US$/h*
*N-3*	Industry	333’922.495,00	6,37
*N-3*	Commerce	2.062’538.316,86	16,04
*N-3*	Private services	86’793.655,29	2,72
*N-3*	Public services	318’689.231,29	13,47
*N-3*	Building	25’664.273,14	8,95
*N-3*	Agriculture & livestock	3’497.251,71	0,11

Sources: 2010 Economic Census.

The grammar was implemented in the accountability MuSIASEM system, which is extremely useful for the study of water metabolism, as it allows to address the multidimensionality of water and to connect nonequivalent definitions of performance through ecologic and social narratives [29]. Specifically, the grammar of water allowed for the integration of relevant information of an internal or social vision, in which water is defined as an ensemble of social flows, and the external or ecosystemic vision in which water is defined as a fund. In this way, useful quantitative information was generated in order to assess the viability of the metabolic pattern of water. The integrated group of semantic categories of accountability of the grammar of water [50] was adapted to the specific characteristics of this study, generating then a quantitative characterization both relevant to water appropriation (within society and ecosystems) as its end use (in society). This study presents generic categories, such as water for homes and economy, differenced from end uses of water. This flexibility in the definition of an accountability outline allowed for data entry to be regulated for the representation. Despite this, the viability of the metabolic pattern in society depends on its compatibility and integrity with external limitations [30, 47, 50]. Therefore, the stability of social appropriation of water was analyzed in quantitative terms, both at a supply level (water extraction) and at the usage or social appropriation of water level. This information showed that the metabolic pattern of Cuenca could be a threat to wild and native ecosystems. Nevertheless, more studies are needed in this area [30, 35, 50].

## Conclusions

This research represents an initial approximation to water studies, its use, and social metabolism in Cuenca, revealing the need to evolve towards interdisciplinarity that connects analysis focused on society and ecosystems. It must be understood that social and ecosystem metabolisms are two different, yet connected processes [3]. This work contributes toward the definition of an analytic framework to assess the metabolism of water within the socio-ecosystems of Cuenca, while establishing a conceptual proposal and methodological tools that can identify scale-based problems and integrate narratives. Our results address an analytical framework that identifies relevant factors that are relevant for the integrated management of hydric resources adapting this outline to specific conditions of water and defining water metabolism of the socio-ecosystem of this Andean canton.

We have used a MuSIASEM framework so as to analyze the water metabolism of Cuenca´s territory. In the context of this article, we have framed the sustainability discussion of metabolic patterns in the use of water in Cuenca to its feasibility. We have found that, due to the biophysical and ecological constraints of each one of Cuenca’s watersheds, Cuenca’s water metabolism is not feasible. We have not analyzed its viability (compatibility with processes under human control) and its desirability (compatibility with normative values and institutions). Further research sustainability discussion of Cuenca’s societal and water metabolism, should be framed within its constraints: ecological constraints (feasibility), technical constraints (viability), and institutional-normative constraints (desirability) [47].

This research represents an approach between studies on hydrology and social metabolism, identifying the need to evolve towards interdisciplinarity of studies that address metabolism and water science that could connect analysis focused on society as part of ecosystems [1]. This work contributes to the definition of this analytical framework of Water Metabolism of Socio-Ecosystems in Cuenca establishing connections formed by a conceptual proposal and a set of methodological tools. In this way, we identified that the metabolism of water in Cuenca’s society shows a strong tendency toward unsustainability although the availability of Social Water Appropriation widely exceeds the Net Use of Water as demonstrated by a relatively low metabolic rate. Industrial and agribusiness uses were highly inefficient, both from a monetary economic logic and from a metabolic logic. Water consumption in urban and rural homes indicates predominantly an unsustainable appropriation of water in Cuenca, with excessively high consumption per person and per day, as well as metabolic rates.

Therefore, it is fundamental to develop policies that guarantee life-water and citizen-water without threatening ecologic metabolism and establishing a connection between social dynamics and ecosystems, and relating this connection to water flows. Rain harvesting and industrial water recycling could significantly reduce the pressure over Cuenca´s rivers. It is essential to reconceptualize water tariffs in order to incentivize water savings and penalize water wasting.

## Supporting information

S1 Table Percentage of the number of grants for each sub-basin and the water volume designed for each sector or water-consuming social system in Cuenca. (DOCX)

S1 File (XLSX)

S2 File (XLSX)

S3 File (XLSX)

S4 File (ZIP)

S5 File (ZIP)

S6 File (RAR)

S7 File (ZIP)
