# Supplementary material for: Looking inside the Blackbox: Cuenca’s water metabolism
Source: PLoS One. 2022 Sep 22;17(9):e0273629. doi: 10.1371/journal.pone.0273629 (PMC9499241; doi:10.1371/journal.pone.0273629)
Supplement: S1 Table — (DOCX) [file pone.0273629.s001.docx]

S1 Table. Percentage of the number of grants for each sub-basin and the water volume designed for each sector or water-consuming social system in Cuenca.

| **Sub-basin** | **Agriculture & livestock** | | **Industry** | | | **Homes** | | **Hydroelectricity** | |
| --- | --- | --- | --- | --- | --- | --- | --- | --- | --- |
|  | **Number of grants (%)** | **Volume (%)** | **Number of grants (%)** | | **Volume (%)** | **Number of grants (%)** | **Volume (%)** | **Number of grants (%)** | **Volume (%)** |
| Tomebamba | 16 | 19.2 | 34.8 | 39.2 | | 12.6 | 74 | 20 | 5.5 |
| Yanuncay | 10.6 | 9.7 | 15.2 | 14 | | 6.2 | 12.6 | 20 | 8.8 |
| Machángara | 9.4 | 30.1 | 28.8 | 45.7 | | 8.5 | 3.8 | 60 | 85.7 |
| Tarqui | 43.8 | 36.2 | 16.7 | 0.4 | | 33.5 | 7.5 | 0 | 0 |
| Cuenca | 13.9 | 3.9 | 1.5 | 0.01 | | 25.6 | 1.3 | 0 | 0 |
| **Total** | **93.7** | **99.1** | **97** | **99.31** | | **86.4** | **99.2** | **100** | **100** |
