# Supplementary material for: Looking inside the Blackbox: Cuenca’s water metabolism
Source: PLoS One. 2022 Sep 22;17(9):e0273629. doi: 10.1371/journal.pone.0273629 (PMC9499241; doi:10.1371/journal.pone.0273629)

EVOLUCION DE IANC 1992 - 2013 (Junio)

SISTEMAS TOMEBAMBA Y MACHÁNGARA

DATOS DEL IANC (PROMEDIO POR AÑO PROYECTADO)

Año	PRODUCCION*	CONSUMO*	IANC
1992	33,775,695	18,544,042	45.10%
1993	33,851,299	17,225,549	49.11%
1994	34,086,860	17,047,511	49.99%
1995	33,845,953	17,484,267	48.34%
1996	34,829,610	18,880,603	45.79%
1997	50,680,784	21,155,527	58.26%
1998	47,359,260	22,116,768	53.30%
1999	46,976,421	21,313,254	54.63%
2000	48,044,385	22,280,666	53.62%
2001	45,696,305	22,231,733	51.35%
2002	41,863,791	22,235,276	46.89%
2003	41,553,281	22,247,942	46.46%
2004	38,226,023	23,011,888	39.80%
2005	36,941,645	23,830,469	35.49%
2006	36,659,449	24,527,924	33.09%
2007	35,412,351	24,482,419	30.86%
2008	35,841,634	25,155,570	29.81%
2009	36,549,480	26,063,976	28.69%
2010	37,226,117	26,598,778	28.55%
2011	37,226,117	26,598,778	28.55%
2012	38,845,733	28,628,036	26.30%
2013	38,573,088	28,650,507	25.72%

* Año 2013 a mayo

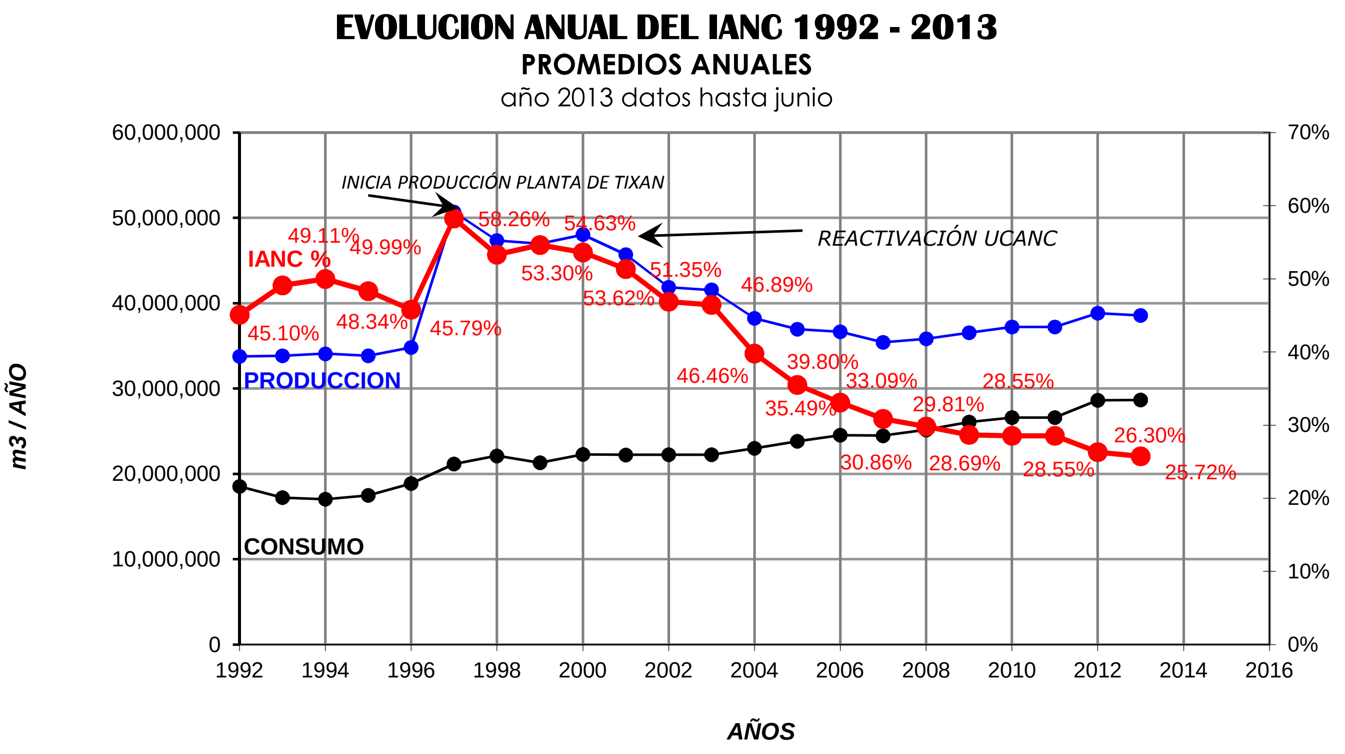

Supplement: S5 File — (ZIP) [file pone.0273629.s006.zip › 2013/evolucion__ianc_reportes_junio2013.pdf]
