# Supplementary material for: Looking inside the Blackbox: Cuenca’s water metabolism
Source: PLoS One. 2022 Sep 22;17(9):e0273629. doi: 10.1371/journal.pone.0273629 (PMC9499241; doi:10.1371/journal.pone.0273629)
Supplement: S5 File — (ZIP) [file pone.0273629.s006.zip › 2013/ianc_mensual_reportes_junio2013.pdf]

EVOLUCION DE IANC 2000 - 2013

UCANC

	MES	VOLUMEN m3. / mes.						INDICE DE ANC % CONSUMIDO
		PRODUCCION CEBOLLAR (1)	PRODUCCION TIXAN (2)	PRODUCCION TOTAL T+M A = (1)+(2)	CAUDAL T+M l/s.	CONSUMIDO T+M D	VOLUMEN T+M F	E = (A - D)/A
2007	**enero/2007**	1650721	1456257	3,106,978	1,160	2,054,545		33.87%
	**febrero/2007**	1462930	1350780	2,813,710	1,163	2,020,595		28.19%
	**marzo/2007**	1588219	1483011	3,071,230	1,147	2,018,378		34.28%
	**abril/2007**	1458853	1469696	2,928,548	1,130	1,995,921		31.85%
	**mayo/2007**	1624319	1380148	3,004,467	1,122	2,083,164		30.66%
	**junio/2007**	1522296	1403352	2,925,648	1,129	1,986,946		32.09%
	**julio/2007**	1550853	1421482	2,972,335	1,110	2,024,010		31.91%
	**agosto/2007**	1480259	1394340	2,874,599	1,091	2,045,118		28.86%
	**septiembre/2007**	1457777	1330600	2,788,377	1,076	2,008,849		27.96%
	**octubre/2007**	1539779	1424936	2,964,715	1,107	2,141,814		27.76%
	**noviembre/2007**	1525579	1399165	2,924,743	1,128	2,074,015		29.09%
	**diciembre/2007**	1589460	1447541	3,037,001	1,134	2,029,064		33.19%
2008	**enero/2008**	1,645,188	1,481,487	3,126,674	1,167	2,226,663		28.78%
	**febrero/2008**	1,538,959	1,359,674	2,898,633	1,157	1,999,065		31.03%
	**marzo/2008**	1,634,738	1,451,618	3,086,356	1,152	2,079,924		32.61%
	**abril/2008**	1,582,083	1,394,835	2,976,918	1,149	2,054,308		30.99%
	**mayo/2008**	1,590,777	1,329,647	2,920,425	1,090	1,977,050		32.30%
	**junio/2008**	1,586,430	1,310,836	2,897,266	1,118	2,082,733		28.11%
	**julio/2008**	1,560,030	1,443,359	3,003,389	1,121	2,154,909		28.25%
	**agosto/2008**	1,536,277	1,480,800	3,017,077	1,126	2,083,110		30.96%
	**septiembre/2008**	1,544,138	1,396,145	2,940,283	1,134	2,119,247		27.92%
	**octubre/2008**	1,563,972	1,455,614	3,019,586	1,127	2,168,750		28.18%
	**noviembre/2008**	1,514,793	1,412,612	2,927,405	1,129	2,033,676		30.53%
	**diciembre/2008**	1,574,955	1,452,667	3,027,622	1,130	2,176,134		28.12%
	**enero/2009**	1,584,464	1,469,546	3,054,010	1,140	2,189,139		28.32%
	**febrero/2009**	1,450,942	1,345,672	2,796,614	1,156	2,045,016		26.88%
	**marzo/2009**	1,578,107	1,460,949	3,039,056	1,135	2,144,235		29.44%
	**abril/2009**	1,553,762	1,440,177	2,993,939	1,155	2,163,276		27.74%
	**mayo/2009**	1,596,687	1,479,661	3,076,348	1,149	2,166,759		29.57%
2009	**junio/2009**	1,542,048	1,477,741	2,970,692	1,146	2,141,137		27.92%
	**julio/2009**	1,598,563	1,530,581	3,079,934	1,150	2,153,356		30.08%
	**agosto/2009**	1,596,234	1,475,092	3,075,331	1,148	2,181,875		29.05%
	**septiembre/2009**	1,544,595	1,464,077	3,008,672	1,161	2,229,301		25.90%
	**octubre/2009**	1,565,985	1,547,473	3,113,458	1,162	2,222,104		28.63%
	**noviembre/2009**	1,721,672	1,383,752	3,105,424	1,198	2,176,326		29.92%
	**diciembre/2009**	1,707,683	1,528,319	3,236,002	1,208	2,251,452		30.42%
	**enero/2010**	1,705,378	1,549,733	3,255,111	1,215	2,208,474	2,209,167	32.15%
	**febrero/2010**	1,538,469	1,409,124	2,947,593	1,218	2,131,971	2,131,467	27.67%
	**marzo/2010**	1,739,698	1,543,123	3,282,820	1,226	2,255,495	2,253,791	31.29%
	**abril/2010**	1,661,871	1,453,632	3,115,503	1,202	2,257,658	2,258,523	27.53%
	**mayo/2010**	1,710,005	1,480,412	3,190,416	1,191	2,162,637	2,164,013	32.21%
2010	**junio/2010**	1,642,529	1,396,198	3,038,727	1,172	2,215,999	2,213,309	27.07%
	**julio/2010**	1,690,459	1,491,070	3,181,528	1,188	2,171,891	2,175,922	31.73%
	**agosto/2010**	1,614,099	1,467,559	3,081,658	1,151	2,210,467	2,211,254	28.27%
	**septiembre/2010**	1,537,626	1,344,744	2,882,370	1,112	2,288,673	2,285,425	20.60%
	**octubre/2010**	1,623,583	1,425,480	3,049,063	1,138	2,315,163	2,317,925	24.07%
	**noviembre/2010**	1,521,021	1,518,013	3,039,034	1,172	2,169,050	2,171,513	28.63%
	**diciembre/2010**	1,643,894	1,518,399	3,162,293	1,181	2,211,300	2,214,194	30.07%
	**enero/2011**	1,674,154	1,555,513	3,229,667	1,206	2,258,870	2,261,866	30.06%
	**febrero/2011**	1,460,032	1,324,876	2,784,907	1,151	2,255,240	2,258,841	19.02%
	**marzo/2011**	1,694,086	1,482,720	3,176,806	1,186	2,235,508	2,236,085	29.63%
	**abril/2011**	1,714,594	1,375,628	3,090,222	1,192	2,198,621	2,200,789	28.85%
	**mayo/2011**	1,730,760	1,406,837	3,137,597	1,171	2,227,006	2,233,081	29.02%
2011	**junio/2011**	1,727,918	1,437,471	3,165,390	1,221	2,285,564	2,286,708	27.80%
	**julio/2011**	1,755,129	1,455,549	3,210,678	1,199	2,204,243	2,218,132	31.35%
	**agosto/2011**	1,746,145	1,428,714	3,174,859	1,185	2,271,467	2,272,407	28.45%
	**septiembre/2011**	1,710,407	1,416,567	3,126,974	1,206	2,256,404	2,255,932	27.84%
	**octubre/2011**	1,792,223	1,480,690	3,272,913	1,222	2,340,357	2,349,284	28.49%
	**noviembre/2011**	1,830,215	1,369,060	3,199,275	1,234	2,321,538	2,322,084	27.44%
	**diciembre/2011**	2,084,319	1,158,764	3,243,083	1,211	2,241,821	2,245,917	30.87%
	**enero/2012**	2,153,955	1,122,343	3,276,298	1,223	2,361,344	2,345,648	27.93%
	**febrero/2012**	2,109,717	1,099,674	3,209,392	1,281	2,420,994	2,424,533	24.57%
	**marzo/2012**	2,147,617	1,160,055	3,307,672	1,235	2,384,059	2,383,647	27.92%
	**abril/2012**	2,020,150	1,099,951	3,120,101	1,204	2,381,781	2,392,186	23.66%
	**mayo/2012**	2,124,882	1,218,791	3,343,673	1,248	2,383,438	2,375,020	28.72%
2012	**junio/2012**	2,062,859	1,102,872	3,165,731	1,221	2,382,403	2,376,513	24.74%
	**julio/2012**	2,090,091	1,132,674	3,222,765	1,203	2,239,648	2,242,876	30.51%
	**agosto/2012**					2,100,897	2,099,983	
	**septiembre/2012**					2,204,270	2,206,415	
	**octubre/2012**	2,079,795	1,104,864	3,184,659	1,189	2,400,081	2,395,344	24.64%
	**noviembre/2012**	1,978,566	1,121,330	3,099,896	1,196	2,427,247	2,423,450	21.70%
	**diciembre/2012**	2,081,854	1,132,199	3,214,052	1,200	2,494,428	2,496,379	22.39%
	**enero/2013**	2,141,388	1,159,574	3,300,962	1,232	2,327,134	2,323,011	29.50%
	**febrero/2013**	1,982,775	1,162,731	3,145,506	1,300	2,602,779	2,598,168	17.25%
2013	**marzo/2013**	2,148,942	1,212,200	3,361,142	1,255	2,506,323	2,523,434	25.43%
	**abril/2013**	2,110,998	1,168,489	3,279,487	1,265	2,504,090	2,498,174	23.64%
	**mayo/2013**	2,095,682	1,270,632	3,366,314	1,257	2,416,324	2,412,296	28.22%
	**junio/2013**	2,006,397	1,188,039	3,194,435	1,232	2,390,648	2,387,181	25.16%

**PROMEDIO 2007**	1,537,587	1,413,442	2,951,029	1,125	2,040,202		30.81%
**PROMEDIO 2008**	1,572,695	1,414,108	2,986,803	1,134	2,096,297		29.82%
**PROMEDIO 2009**	1,586,728	1,466,920	3,045,790	1,159	2,171,998		28.66%
**PROMEDIO 2010**	1,635,719	1,466,457	3,102,176	1,181	2,216,565	2,217,209	28.44%
**PROMEDIO 2011**	1,743,332	1,407,699	3,151,031	1,199	2,258,053	2,261,761	28.24%
**PROMEDIO 2012 (hasta junio)**	2,103,197	1,133,948	3,237,144	1,235	2,385,670	2,382,925	26.26%
**PROMEDIO 2012**	2,084,949	1,129,475	3,214,424	1,220	2,387,542	2,385,560	25.68%
**PROMEDIO 2013**	2,081,030	1,193,611	3,274,641	1,257	2,457,883	2,457,044	24.87%
**PROMEDIO ULTIMO TRIMESTRE**	2,071,025	1,209,053	3,280,079	1,251	2,437,021	2,432,550	25.68%
