# Supplementary material for: Looking inside the Blackbox: Cuenca’s water metabolism
Source: PLoS One. 2022 Sep 22;17(9):e0273629. doi: 10.1371/journal.pone.0273629 (PMC9499241; doi:10.1371/journal.pone.0273629)
Supplement: S7 File — (ZIP) [file pone.0273629.s008.zip › Base_de_Datos_ESPAC_2006-SPSS.zip!/BASE_DE_DATOS.pdf]

BASE DE DATOS DE SÍNTESIS

ENCUESTA DE SUPERFICIE Y PRODUCCIÓN AGROPECUARIA CONTINUA

ESPAC

Base de Datos Sintética (BDD), conocida también como BDD de Resumen, tratándose en este sentido de un versión abreviada de los datos expandidos (Estimaciones) a nivel agregativo provincial, mostrándose como un conjunto exhaustivo no redundante de datos estructurados organizados independientemente de su utilización y su implementación.

El presente producto, se caracteriza principalmente por definirse como un tipo de base de datos Flot-File o tipo Excel, donde todos los datos se encuentran relacionados entre ellos situados en una única tabla.

A continuación, se detalla claramente la descripción de las BDD de la Encuesta de Superficie y Producción Agropecuaria Continua ESPAC, definida por capítulos, siendo estos:

**Nombre Base de Datos**	**Descripción Base de Datos**
USO(*A)	Capítulo 3. Características del uso del suelo de la UPA
CULTIVOS(*A)P	Capítulo 4. Cultivos Permanentes de la UPA
CULTIVOS(*A)T	Capítulo 5. Cultivos Transitorios de la UPA
DISPERSOS(*A)	Capítulo 6. Árboles o plantas permanentes dispersos de la UPA
FLORES(*A)	Capítulo 7. Floricultura de la UPA
VACUNO(*A)	Capítulo 8. Ganado Vacuno de la UPA
PORCINO(*A)	Capítulo 9. Ganado Porcino de la UPA
OVINO(*A)	Capítulo 10. Ganado Ovino de la UPA
OESPECIES(*A)	Capítulo 11. Otras especies de ganado de la UPA
AVES(*A)	Capítulo 12. Aves de campo y de planteles avícolas de la UPA
EMPLEO(*A)	Capítulo 13. Empleo de la UPA

(*A) Especifica el año de la encuesta.

Cabe señalar, que el formato de las bases se encuentra en .TXT (Delimitado por tabulaciones) con estándar ANSI, lo que permite al usuario final una fácil migración y

lectura de los archivos en cualquier programa utilitario como: Wordpad, Excel, Word; y/o programa estadístico: SAS, SPSS, Gauss, Matlab, Stata, JMP; y/o bases de datos como Access, FoxPro; entre otros.

No se provee de la documentación típica que siempre acompaña a las bases de datos como: Diccionario de Datos (DDD), Metodología; ya que cada base esta completamente etiquetada y desagregada al nivel permisible, que coadyuva al mejor dinamismo en la preparación de tablas de resultados y trabajo de consulta en las bases.

INEC - ESAG
